# The Rate of Postoperative Bile Leak in Minimally Invasive Liver Resection in Comparison With Open Surgery: A Systematic Review

**DOI:** 10.7759/cureus.74313

**Published:** 2024-11-23

**Authors:** Eiad Elmahi, Samantha Fairclough, Harry Knifton

**Affiliations:** 1 General Surgery, Lincoln County Hospital, Lincoln, GBR; 2 General Surgery, North West Anglia NHS Foundation Trust, Peterborough, GBR; 3 General Surgery, University Hospitals of Leicester NHS Trust, Leicester, GBR

**Keywords:** hepatectomy, hepatobiliary surgery, laparoscopic liver resection, open liver resection, post-operative complications

## Abstract

The rapid advances in laparoscopic surgery have meant that formerly complex techniques are now commonly performed via this method. These practices are now becoming increasingly popular in the discipline of hepatopancreaticobiliary (HPB) surgery. One such example is liver resection, which is the focus of our review. We aimed to assess the rate of bile leak complications in minimally invasive liver resection compared to an open liver approach in malignant and benign conditions. A systematic review spanning the period from 2000 to 2022 was conducted, examining the postoperative complications in laparoscopic versus open liver resections. We searched the databases Medline, Cochrane, PubMed, and Google Scholar for relevant studies; 16 studies were included in the final analysis.

Ten out of 16 studies that were included indicated that there was no significant difference in the rate of bile leaks. Five studies showed that bile leaks were found to occur more frequently in open surgery, and one study suggested that the rates were more common with the laparoscopic approach. The overall comparison of bile leak rates following open and minimally invasive liver resection suggests that there is no reduction in this complication in both types of surgery. As such, a laparoscopic or open method can both be adopted without any concerns for this particular complication.

## Introduction and background

The advent of the laparoscopic technique has revolutionised operative practice across surgical disciplines. Although several reviews have demonstrated improved postoperative outcomes for patients who undergo laparoscopic surgery, the practice has been slow to take off in hepatopancreaticobiliary (HPB) surgery. This is particularly notable in liver resection where there is a scarcity of literature examining outcomes between the laparoscopic and open approach. However, based on the research that has been published, there is demonstrable evidence to suggest that the laparoscopic technique can reduce the rate of postoperative complications [1,2].

There are several reasons why a liver resection may be clinically indicated, with liver metastasis being the most common [3]. Another important indication is hepatocellular carcinoma (HCC), the most common primary malignancy of the liver, which is associated with one of the highest mortality rates among cancers [4]. Benign neoplasms of the liver may also require management with hepatectomy, the most common being haemangioma - though operative management is not usually indicated unless complications are encountered [5]. Adenomas, lipomas, and fibromas are other benign causes that tend to be asymptomatic unless complications manifest [6]. Regardless of the surgical approach and indeed the clinical indication, liver resection itself remains a major operation. Complications that patients should be informed of in the consent process include haemorrhage, infection, injury to surrounding structures, and bile leak.

Despite advances in perioperative care and surgical technique, bile leak remains a significant complication of hepatectomy. It can have a serious impact on the postoperative course of the patient as it is associated with the development of sepsis, liver failure, and prolonged hospital admission [7]. The management of postoperative bile leak will vary depending on the severity of the complication but may require interventional management in the form of percutaneous drain insertion or endoscopic retrograde cholangiopancreatography (ERCP) [8]. When operative intervention is indicated, a laparoscopic approach is often preferred [8].

Though there is generally scarce data regarding the comparison of open and laparoscopic approaches in liver resection, a recent systematic review led by our first author [2] compared the amount of blood loss in laparoscopic versus open hepatectomy. Eleven of the 12 studies that Elmahi et al. [2] examined demonstrated reduced blood loss in minimally invasive approaches, which led them to conclude that laparoscopic surgery could be the preferred approach for both benign and malignant conditions. Building upon Elmahi et al.'s review of blood loss, we have collated the postoperative complications of laparoscopic liver resection (LLR) with those of open liver resection (OLR) as documented in the current literature to examine the rates of bile leak between the two approaches.

We believe our findings may help establish a standard of practice for minimally invasive liver resection surgery. Also, a comparative analysis of these two approaches may contribute to major changes in surgical practice, which will reduce the sequelae of complications from bile leaks as well as the burden of the associated cost [9].

## Review

Materials and methods

Search Strategy

**Figure 1:**
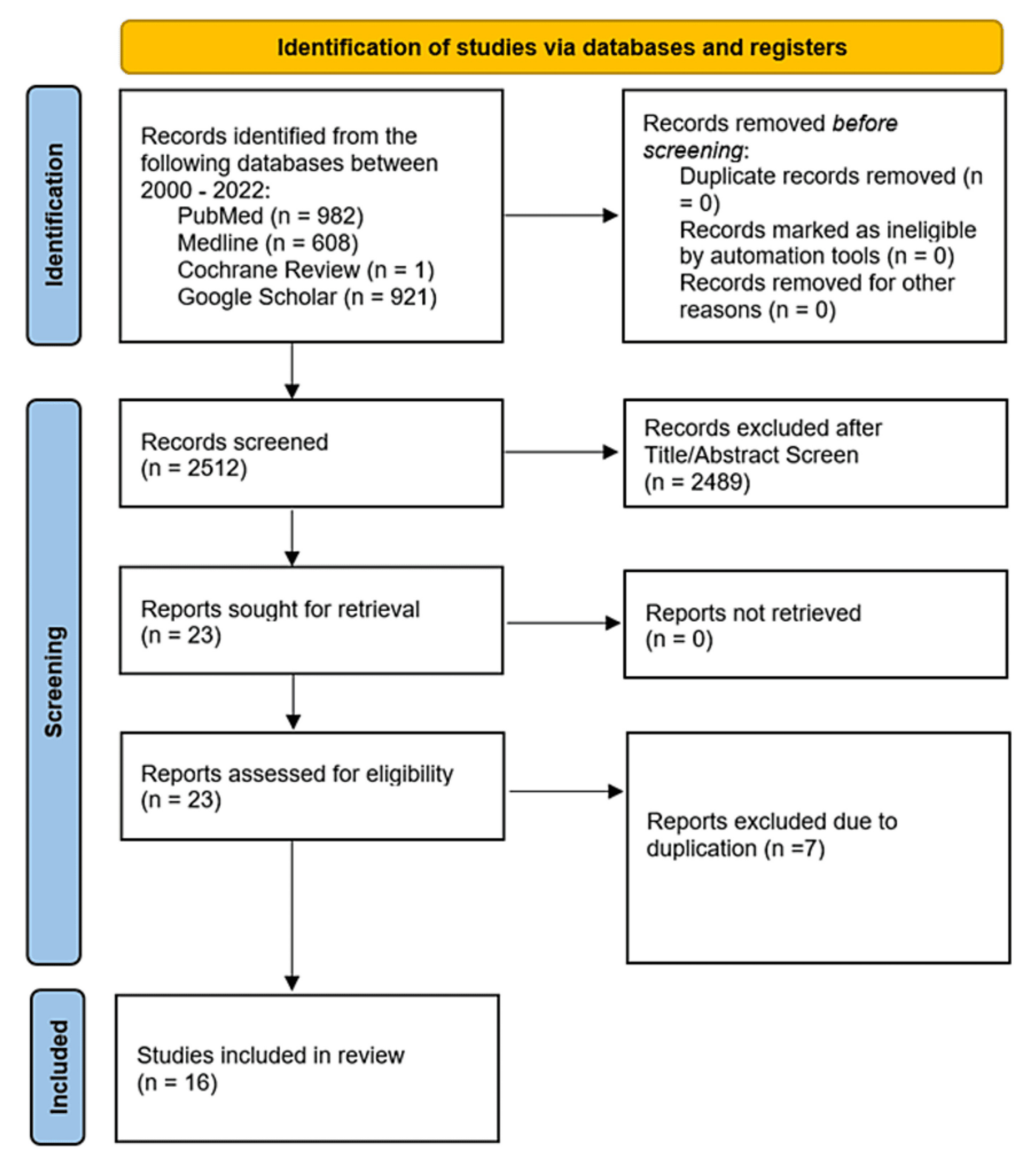
PRISMA diagram depicting the search strategy PRISMA: Preferred Reporting Items for Systematic Reviews and Meta-Analyses

In this systematic review, we aimed to compare the postoperative rate of bile leak complications between laparoscopic and open liver resection, spanning the period from 2000 to 2022. We conducted a literature search based on the Preferred Reporting Items for Systematic Reviews and Meta-Analyses (PRISMA) guidelines (Figure 1), involving the databases of Cochrane, Google Scholar, Medline, and PubMed, to identify relevant papers that met our inclusion criteria.

Research Protocol

Studies comparing bile leak complications between laparoscopic and open approaches were identified and then examined based on our inclusion and exclusion criteria. Identification, data extraction, quality assessment, and statistical analyses of the respective studies were included in the research protocol. The PICO (population, intervention, control, and outcomes) method was employed to select the most relevant studies in the period from 2000 to 2022.

Inclusion Criteria

The inclusion criteria in our systematic review were as follows: studies comparing bile leak complications between laparoscopic and open approaches to liver resection; a specified indication for each surgery; and the type of procedure and other postoperative complications occurring as a direct consequence of both techniques.

Exclusion Criteria

Studies that did not contain a reported outcome were excluded, along with those where metastatic disease was the indication for liver resection. Studies not published in English were also excluded.

Study Selection and Quality Assessment

The quality and reliability of the publications selected for review were assessed using the Effective Public Health Practice Project (EPHPP).

Data Extraction and Analysis

A data extraction form was employed to address the relevant questions in our systematic review. Guidelines from the World Health Organisation (WHO) were also referred to when assessing complications of liver resection.

Results

Literature Search

Initially, 2512 publications were identified via the literature search (Table 1). Of these, 2489 studies were excluded because they were irrelevant or not comparative; 23 studies met the inclusion criteria, and seven were excluded as they were duplicates. Ultimately, 16 studies were included in the final analysis. We did not identify any randomised control trials (RCT) comparing the rate of bile leak complication in laparoscopic and open resection. Table 2 provides a summary of the 16 studies selected.

**Table 1:** Search terminologies used

PubMed search, accessed on 20 October 2022 (926 articles)	Medline search, accessed on 20 October 2022 (605 articles)	Cochrane Reviews search, accessed on 20 October 2022 (1 article)	Google Scholar search, accessed on 20 October 2022 (921 articles)
(laparoscopic hepatectomy OR minimally invasive) AND (post-operative complications OR post-hepatectomy bile leak OR intra-operative bile leak) AND (open liver resection) AND (versus AND compare) OR (hepatobiliary surgery AND segmentectomy)	(laparoscopic hepatectomy OR minimally invasive) AND (post-operative complications OR post-hepatectomy bile leak OR intra-operative bile leak) AND (open liver resection) AND (versus AND compare) OR (hepatobiliary surgery AND segmentectomy)	(laparoscopic hepatectomy OR minimally invasive) AND (post-operative complications OR post-hepatectomy bile leak OR intra-operative bile leak) AND (versus AND compare)	(laparoscopic hepatectomy OR minimally invasive) AND (post-operative complications OR post-hepatectomy bile leak OR intra-operative bile leak) AND (open liver resection) AND (versus AND compare) OR (hepatobiliary surgery AND segmentectomy)

**Table 2:** Summary of the included studies LLR: laparoscopic liver resection; OLR: open liver resection

Author(s)	Year	Location	Study type	Number of patients	LLR/OLR	Eligibility for inclusion	Quality assessment	Ethical appraisal
Zhang et al. [1]	2015	China	Cohort study	50	30/20	Yes	Yes	Yes
Shimada et al. [10]	2001	Japan	Comparative matched	55	17/38	Yes	Yes	Yes
Laurent et al. [11]	2003	France	Comparative matched	27	13/14	Yes	Yes	Yes
Mala et al. [12]	2002	Germany	Comparative matched	27	13/14	Yes	Yes	Yes
Tee et al. [13]	2020	China	Cohort study	1769	487/1282	Yes	Yes	Yes
Buell et al. [14]	2004	USA	Comparative matched	117	17/100	Yes	Yes	Yes
Koffron et al. [15]	2007	USA	Comparative matched	300	241/59	Yes	Yes	Yes
Aldrighetti et al. [16]	2008	Italy	Comparative matched	40	20/20	Yes	Yes	Yes
Polignano et al. [17]	2008	UK	Comparative matched	50	25/25	Yes	Yes	Yes
Carswell et al. [18]	2009	UK	Comparative matched	20	10/10	Yes	Yes	Yes
Hu et al. [19]	2011	China	Comparative matched	60	30/30	Yes	Yes	Yes
Slakey et al. [20]	2013	USA	Retrospective review	62	45/17	Yes	Yes	Yes
Nassar et al. [21]	2015	Egypt	Comparative matched	30	15/15	Yes	Yes	Yes
Smith et al. [22]	2020	USA	Case-control	1388	599/789	Yes	Yes	Yes
Barba et al. [23]	2022	Italy	Cohort study	170	85/85	Yes	Yes	Yes
Qiu et al. [24]	2014	USA	Comparative matched	49	24/25	Yes	Yes	Yes

Comparison of Bile Leak Rates Between OLR and LLR

The rate of postoperative bile leak was compared in the LLR and OLR groups to assess the outcomes of the different approaches (Table 2). Shimada et al. [10] studied 38 patients who had undergone LLR versus 17 patients who had OLR for liver malignancy. No significant difference in bile leak (p=0.89) was found between the groups. Laurent et al. [11] reported similar results in their study. They compared 13 patients who underwent LLR to 14 patients who had OLR, and the difference in bile leak was not statistically significant.

Although the bile leak rate was found to be more prominent in the open versus laparoscopic approach in the study by Mala et al. [12], this was not statistically significant. This particular study enrolled 27 patients, 13 of them operated laparoscopically while the rest (14) were managed conventionally. Two patients who had undergone open surgery experienced a leak in comparison to zero patients who experienced a leak after the laparoscopic approach. On the contrary, Tee et al. [13] reported bile leak rates of 3.7% and 9.7% in the minimally invasive group and open hepatectomy respectively (p=0.001).

Buell et al. [14] compared the outcomes of 17 LLRs vs. 100 OLRs for non-malignant tumours, with one patient needing to go back to theatre for a bile leak in the OLR group (p<0.05). Koffron et al. [15] evaluated 300 liver resections: 241 LLR; 59 OLR and laparoscopic assisted. This study found a bile leak rate of 2.7% versus 4% in laparoscopic and open respectively, all of which did not require any operative intervention; the difference was not significant. Aldrighetti et al. [16] compared 20 LLRs with 20 OLRs with one bile leak in the open group versus zero in the laparoscopic group (p<0.05). 

The studies by Polignano et al. [17] and Carswell et al. [18] did not show a difference in bile leak rates in either group. The former included 50 patients (25 LLR and 25 OLR) and the latter included 10 LLR and 10 OLR. On the other hand, Hu et al.'s study [19], which compared 30 minimally invasive hepatectomies to 30 open hepatectomies showed a contrasting pattern. Three patients in the minimally invasive group had bile leaks from a transected liver surface, requiring drains for three days, in comparison to the open group which had no patients with bile leaks postoperatively.

Slakey et al. [20] found that one patient in the open group had a leak versus none in the laparoscopic group (p=0.61). This study reviewed 62 patients: 45 LLR and 17 OLR. Nassar et al.'s [21] study followed a different trend from Slakey et al. [20]. This study compared 15 LLR to 15 OLR with one patient found to have a leak in the LLR group in comparison to the two patients in the OLR group (p=0.49). Smith et al. [22] endorsed the findings of Slakey et al.'s [20] study, which involved a multi-institutional case-control study of 1388 resections and showed a non-statistically significant reduced bile leak rate in patients in the LLR versus OLR group (p=0.21). They compared 599 LLR against 789 and found that 26 patients experienced a leak postoperatively in the open group versus 13 in the laparoscopic group. Another similar study by Barba et al. [23] looked at 85 minimally invasive liver resections versus 85 open liver resections. The bile leak rate was found to be 2% and 10% respectively (p=0.015).

Qiu et al.'s study [24] involved a comparative analysis of 49 patients: 24 LLR and 25 OLR. Their results showed that one patient in the LLR group had a leak in comparison with no patients in the OLR, although this was found not to be significant. Similarly, in the study by Zhang et al. [1], which compared 30 LLR to 20 OLR, there was no significant difference in the rate of leaks in either group: one patient in the laparoscopic group versus no patients in the open group.

Discussion

A minimally invasive approach in hepatobiliary surgery has always been controversial due to a lack of high-quality evidence supporting the superiority of this approach to the conventional technique [11]. This systematic review was conducted to assess the clinical efficiency and the postoperative complications of laparoscopic liver resection compared to the classic technique concerning bile leaks. It is important to note that at present there are no published randomised controlled trials comparing these two techniques.

Although many surgeons claim that using the laparoscopic approach in liver resection is challenging due to technical issues such as the rigidity of instruments, a narrow operative field, and the complexity of peripheral liver tumour resections [25, 26], others believe that it is a feasible technique for hepatectomies, as laparoscopic instruments and training have significantly improved in the last three decades [15]. Therefore, we believe that it should be considered the gold standard of treatment for the majority of hepatectomies.

The studies reviewed here have indicated that there is no significant reduction in the rates of postoperative bile leak rates with a laparoscopic approach overall. Ten of the studies concluded that neither approach reduced the rates of a leak; five studies were in favour of the laparoscopic method and one paper favoured the open approach. As such, it is no surprise that there is an ongoing dispute as to which method is superior, with some surgeons still favouring the conventional approach. There may be other factors that influence the decision to choose one approach over another regarding postoperative complications; however, the rate of bile leak does not appear to be a convincing factor when considering this specific postoperative complication.

Limitations

Although this systematic review included high-quality studies, we could not identify any RCTs comparing the outcome of LLR and OLR at present. Moreover, although patients included in this review have been operated on by experienced surgeons, the level of experience varies among consultant surgeons.

Implications of This Review on Current Practice

Based on our findings, there is no evidence to suggest that a minimally invasive approach is clinically more efficient and associated with fewer postoperative complications than an open approach. Hence, we recommend that our findings be taken into account as part of a wider perspective in the context of other postoperative complications before determining which method to adopt. 

## Conclusions

Based on our review of the current literature, there is no established benefit of reduced rates of bile leak postoperatively in LLR versus OLR. As discussed, we did not take into account other specific complications such as blood loss, length of stay in the hospital, or postoperative pain, among other factors. Higher quality studies such as RCTs are required to ensure an accurate comparison to find out if one surgical approach is superior to another. This review found that the rate of bile leak postoperatively is not a reliable measure to assess the superiority of minimally invasive techniques.
